# Two-photon scanned light sheet fluorescence microscopy with axicon imaging for fast volumetric imaging

**DOI:** 10.1117/1.JBO.26.11.116503

**Published:** 2021-11-18

**Authors:** Po-Yen Lin, Sheng-Ping L. Hwang, Chi-Hon Lee, Bi-Chang Chen

**Affiliations:** a Institute of Cellular and Organismic Biology, Academia Sinica, Taipei, Taiwan; b Research Center for Applied Sciences, Academia Sinica, Taipei, Taiwan

**Keywords:** three-dimensional imaging, two-photon microscopy, light sheet fluorescence microscopy

## Abstract

**Significance:** Two-photon microscopy has become the standard platform for deep-tissue fluorescence imaging. However, the use of point scanning in conventional two-photon microscopy limits the speed of volumetric image acquisition.

**Aim:** To obtain fast and deep volumetric images, we combine two-photon light sheet fluorescence microscopy (2p-LSFM) and axicon imaging that yields an extended depth of field (DOF) in 2p-LSFM.

**Approach:** Axicon imaging is achieved by imposing an axicon lens in the detection part of LSFM.

**Results:** The DOF with axicon imaging is extended more than 20-fold over that of a conventional imaging lens, liberating the synchronized scanning in LSFM. We captured images of dynamic beating hearts and red blood cells in zebrafish larvae at volume acquisition rates up to 30 Hz.

**Conclusions:** We demonstrate the fast three-dimensional imaging capability of 2p-LSFM with axicon imaging by recording the rapid dynamics of physiological processes.

## 1 Introduction

Techniques to capture deep volumetric images at high speed are widely applicable in biological research and particularly useful in neuroscience and developmental biology. In neuroimaging, optical signals that respond to neuronal activity can change on millisecond timescales and propagate dozens of micrometers in three dimensions.^1^ Moreover, studies on cardiac development in embryonic zebrafish typically require imaging rates of ∼10 volumes per second (volume acquisition rate of 10 Hz) to capture the 3D dynamics of a heart beating at 2 to 4 Hz.^2^ Imaging such rapid processes deep within tissues is still challenging with current bioimaging techniques. Recently developed techniques for deep-tissue imaging typically utilize two-photon microscopy, which enables the visualization of fluorescence deep inside intact tissues.^3^^,^^4^ In conventional two-photon microscopy, a near-infrared ultrashort pulsed laser is focused by an objective on a particular spot to excite fluorophores within the sample. The fluorescent signal is only generated in this one focal point, and emitted photons can be collected with the same objective and detected with a point detector. After scanning the focal spot throughout the sample, a volumetric image is constructed by stacking a series of optical section images. However, the use of point scanning imposes a fundamental limitation on the volume imaging rate, according to the laser repetition rate and fluorescence lifetime of fluorophores;^5^ with this technique, the integration time per voxel cannot be shorter than ∼10  ns. The volume imaging rate may be increased by reducing the voxel number (skipping those voxels without objects of interest) or by scanning the sample with an extended focal volume.^6^^,^^7^ A major difficulty with skipping voxels is that the positions of targets must be known prior to imaging, and the objects of interest must remain immobile. Otherwise, motion-induced image artifacts will occur. Alternatively, whole-volume scanning by an axially extended focal spot can also be used speed up the volume acquisition rate (up to 50 Hz),^8^ with the excitation focal volume stretched from a Gaussian to a Bessel focus.^9^^–^^11^ The scanning of a single extended focal volume turns a 2D scan into a 3D scan, because each pixel in the x−y image contains an axial projection of elongated focal volume. However, this approach is also limited. Although extended depth of field (DOF) scanning allows one to reduce the number of 2D scans needed to probe an entire volume of interest, axial resolution is also lost. Parallelized detection by a camera is a potential strategy for fast volumetric deep imaging. A two-photon excitation plane can be generated by multifocus scanning,^12^ temporal focus,^13^ or a light sheet.^14^ Within the excitation plane, fluorescent objects may be imaged by a wide-field microscope. In contrast to a point scan approach, camera-based detection reduces the scanned dimensions for volumetric imaging from three (x−y−z scan) to only one (z axial scan). As such, light sheet fluorescence microscopy (LSFM) has recently emerged as a promising imaging platform for biological research. Benefits of LSFM include intrinsic optical sectioning, low phototoxicity, and high spatiotemporal resolution.^15^^,^^16^

In a conventional LSFM, the excitation plane can be generated either statically, using a cylindrical lens,^14^ or dynamically, using a rapidly scanning laser beam.^17^ The thickness of light sheet is the key determinant of optical sectioning capability. Notably, the excitation plane must be carefully aligned to the focal plane of an orthogonally arranged detection objective lens; otherwise, a blurred image will be obtained. In contrast to wide-field microscopy, the combination of excitation and detection point spread functions (PSFs) can improve axial resolution.^18^ In this optical configuration, a 3D volumetric image can be formed by synchronized scanning of the co-aligned plane throughout the sample. Scanning of the detection objective focal plane is usually performed with a piezoscanner. However, fast 3D volumetric imaging with this method is limited by the mechanical motion of the piezocoupled objective. For high-resolution imaging, a high numerical aperture (NA) objective must be used; typically, this type of objective is heavy and will exhibit very shallow DOF. It is still a technical challenge to drive a heavy mass at high speed using a piezoscanner. Moreover, when performing a rapid z scan, objective vibration may cause unwanted artifacts in the image or disturb delicate biological samples. In order to prevent vibration, it would be preferable to keep the sample and the objective immobile, only scanning the light sheet to obtain a 3D image. However, the light sheet will scan outside the DOF of an immobile detection objective, prohibiting 3D imaging. Hence, the use of a detection objective with an extended DOF could keep the image in focus when scanning the light sheet along the z axis of the detection objective. Several methods have been used to extend the DOF detection objectives for fast 3D volumetric imaging, including an electrically tunable lens,^19^^,^^20^ remote focusing,^21^ an acoustic gradient lens,^22^^,^^23^ and a deformable mirror.^24^ These methods prevent vibration from the movement of the detection objective; however, the methods still require synchronization of excitation plane and the focal plane scans, and each may introduce other aberrations. Alternatively, wave-front coding by placing the phase masks in the detection pupil^25^^,^^26^ or oblique plane illumination by reorienting the imaging system^27^^–^^30^ can also extend DOF of the detection objective. Yet these methods require extensive postprocessing or complex implementations, making them difficult to set up and impractical for large datasets. In addition to LSFM, light field microscopy (LFM) also offers fast 3D imaging capability. It reconstructs a 3D image via recording the light field from samples in a single 2D image, but the background noise will inhibit its usability for deep tissue imaging.^31^^,^^32^ Although the background noise can be suppressed to improve LFM performance,^33^^,^^34^ pushing the resolution remains a challenge.

In this paper, we describe a simple method to increase the DOF in a two-color two-photon LSFM system, making it capable of fast 3D deep imaging. To do so, we added a refractive axicon in the imaging part of a two-photon (2p) LSFM system. With this setup, we were able to obtain volumetric 3D images without any need for z scanning with a piezocoupled detection objective. The volumetric imaging speed was only limited by the camera rate and photon budget. Furthermore, the DOF of the imaging objective was extended by ∼20-fold compared to that of a conventional imaging lens, and the setup maintains similar isotropic spatial resolution to standard LSFM. We also demonstrated the fast 3D imaging capability of our system for 2p LSFM with axicon imaging by recording the rapid dynamics of physiological processes. Blood cells and beating hearts of 3 days postfertilization (dpf) larval zebrafish were monitored at volume acquisition rates of 30 and 10 Hz, respectively.

## 2 Methods

**Fig. 1:**
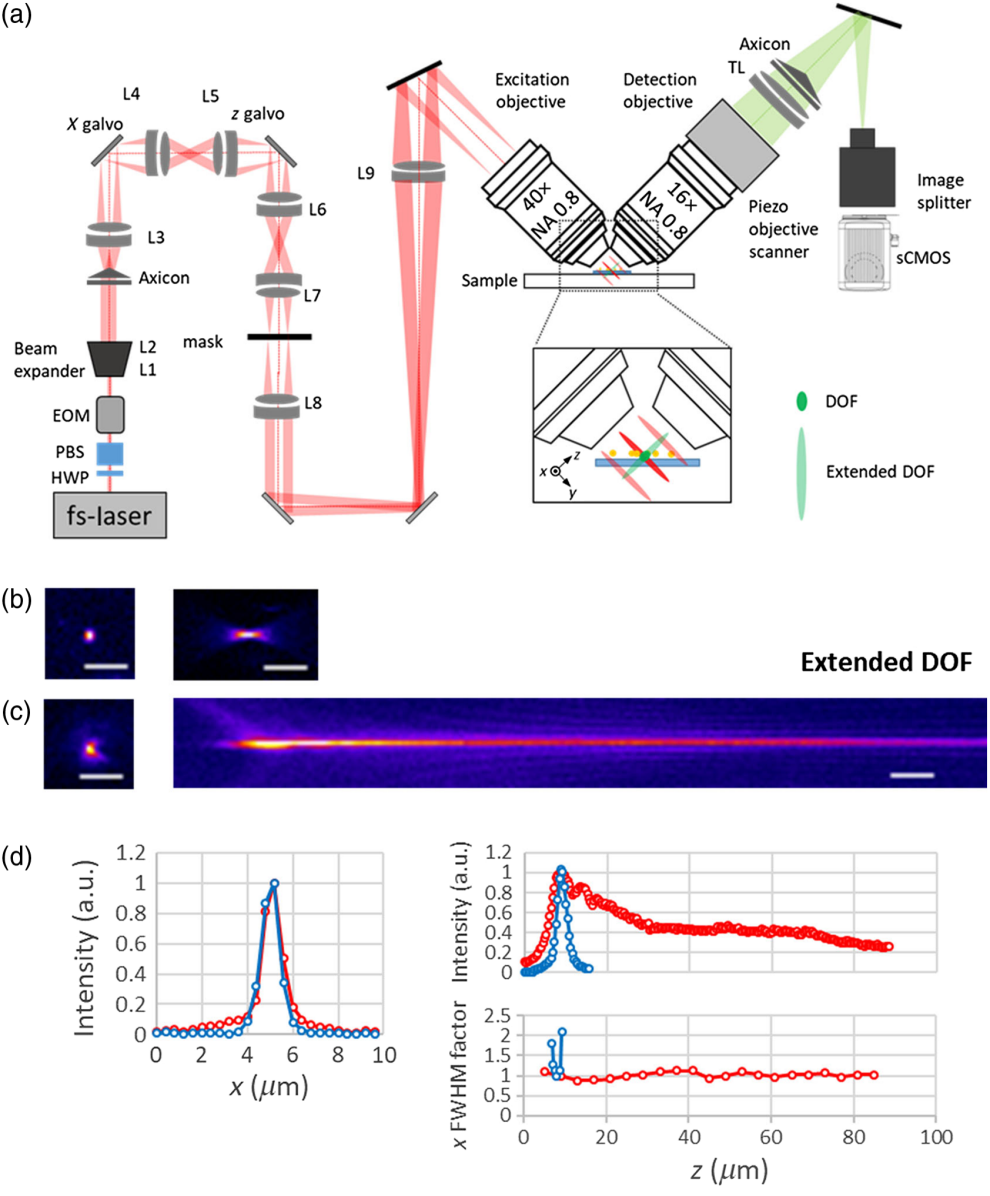
(a) Schematic of 2p LSFM with axicon imaging. Inset shows the extended DOF using axicon imaging. Measured PSFs (b) without and (c) with axicon imaging. (d) The corresponding intensity line profiles of measured PSF and lateral FWHM factor at different axial positions: lateral (left), axial (upper right); lateral FWHM factor (lower right). Red: with axicon imaging. Blue: without axicon imaging. HWP, half-wave plate; PBS, polarization beam splitter; L1 to L9, achromatic doublet lens; and TL, tube lens. Scale bars: 5  μm.

A schematic of the setup for 2p LSFM with axicon imaging (extended DOF) is shown in Fig. 1(a). The two-photon excitation light is provided by a tunable dual output fs-laser system (Coherent, Chameleon Discovery) with 80 MHz repetition rate; one beam is fixed at 1040 nm and a second beam is tunable (680 to 1300 nm). Individual beam powers are adjusted using half-wave plates and polarization beam splitters. For two-color two-photon imaging, the two laser beams must be precisely aligned onto a single optical axis with a dichroic mirror (NFD01-1040-25x36, Semrock) and then conducted to an electro-optical modulator (320RM, Conoptics) to control the laser intensity when imaging.

The laser beam is expanded to ∼15  mm diameter by a telescope [L1 and L2 in Fig. 1(a), AC254-050-B-ML and AC254-250-B-ML, Thorlabs] and then passed through an axicon set (AX2505B, Thorlabs) and a lens [L3 in Fig. 1(a); AC254-150-B-ML, Thorlabs] to convert the Gaussian profile to a Bessel beam. A ring pattern is, therefore, generated and then conjugated to a set of galvanometer scanners (8315K, Cambridge Technology), which are composed of a pair of achromatic lenses [L4 and L5 in Fig. 1(a); AC254-080-B, Thorlabs] in a 4f arrangement and scan the excitation beams in order to produce an illumination light sheet and perform a z axis scan. After passing through the scanning unit, the ring is magnified through a relay lens set (L6 and L7, AC254-060-B and AC254-125-B, Thorlabs) and conjugated to an annular aperture mask as a spatial filter. The inner and outer diameter of the annular aperture are 2.24 and 2.99 mm (outer NA=0.36 and inner NA=0.27 formed in the excitation objective), respectively. A lens pair [L8 and L9 in Fig. 1(a); AC254-250-B and AC254-300-B, Thorlabs] relays the ring onto the back focal plane of the excitation objective (40×, 0.8 NA, Nikon). Fluorescence from the excitation plane is collected by a water-immersion objective (16×, 0.8 NA, Nikon), which is mounted on a piezoscanner (P-726.1CD PIFOC, Physik Instrumente) and placed with its axis orthogonal to the excitation plane. A tube lens (TTL 200, Thorlabs) and an image splitter with YFP and GPF filters sets (W-view, Hamamatsu) are used to create the two-color fluorescence images captured with a scientific complementary metal-oxide-semiconductor camera (Orca Flash 4.0 v2, Hamamatsu). The microscope is controlled as pervious description.^35^ Briefly, all signals are processed by custom LabVIEW software and a field-programmable gate array card (National Instruments, PCIe-7852R). Control signals for the electro-optical modulator, galvo scanners, and the detection-objective piezo are triggered by the imaging camera and conditioned by individual scaling amplifiers (SRS, SIM983, and SIM900 mainframe). In order to extend the DOF in the imaging part of the 2p LSFM system, an axicon (AX252-A, Thorlabs) is placed at 5 cm behind the tube lens and the camera is adjusted to the position where the image is in focus. The axicon can not only elongate the PSF axially, but it also maintains the lateral resolution. The needle-like PSFs are used both in excitation and imaging parts of this 2p LSFM system.

## 3 Results and Discussion

To evaluate the imaging part of our system, 200-nm diameter fluorescent microspheres (Polysciences) were mounted on a 5-mm coverslip and used to measure the PSF of the microscope. Figures 1(b) and 1(c) show the x−y and x−z images acquired from fluorescent beads, without and with axicon, by scanning the detection objective in the z direction. The signal-to-noise ratio of images acquired with the axicon was poor due to strong spherical aberrations induced by the axicon. However, postprocessing techniques, such as deconvolution or image filter processing, may be applied to improve the image quality.^36^^,^^37^ Spherical aberration can also extend the axial extent of the detection PSF,^38^ but the lateral resolution will loss in high NA system.^39^^,^^40^ In Fig. 1(d), the measured PSF with axicon imaging shows an asymmetry distribution, probably due to the imperfections in the axicon apex.^41^ However, the lateral resolution with axicon imaging still remains unchanged for at least 80  μm as comparing the lateral full-width of half-maximum (FWHM) at different axial positions. A circularly symmetric stair step device placed in a pupil plane is another method to achieve extended DOF by exploiting the finite coherence length of emission light.^42^ However, this method offers a finite extension of DOF by a factor of 3 to 5 and a lateral resolution blurring of a factor of 1.2 as comparing to a standard PSF. Thus the elongated DOF of the axicon imaging system has relatively poor axial resolution, but it allows more axial information to be recorded from the image plane as well as maintains the lateral resolution.

**Fig. 2:**
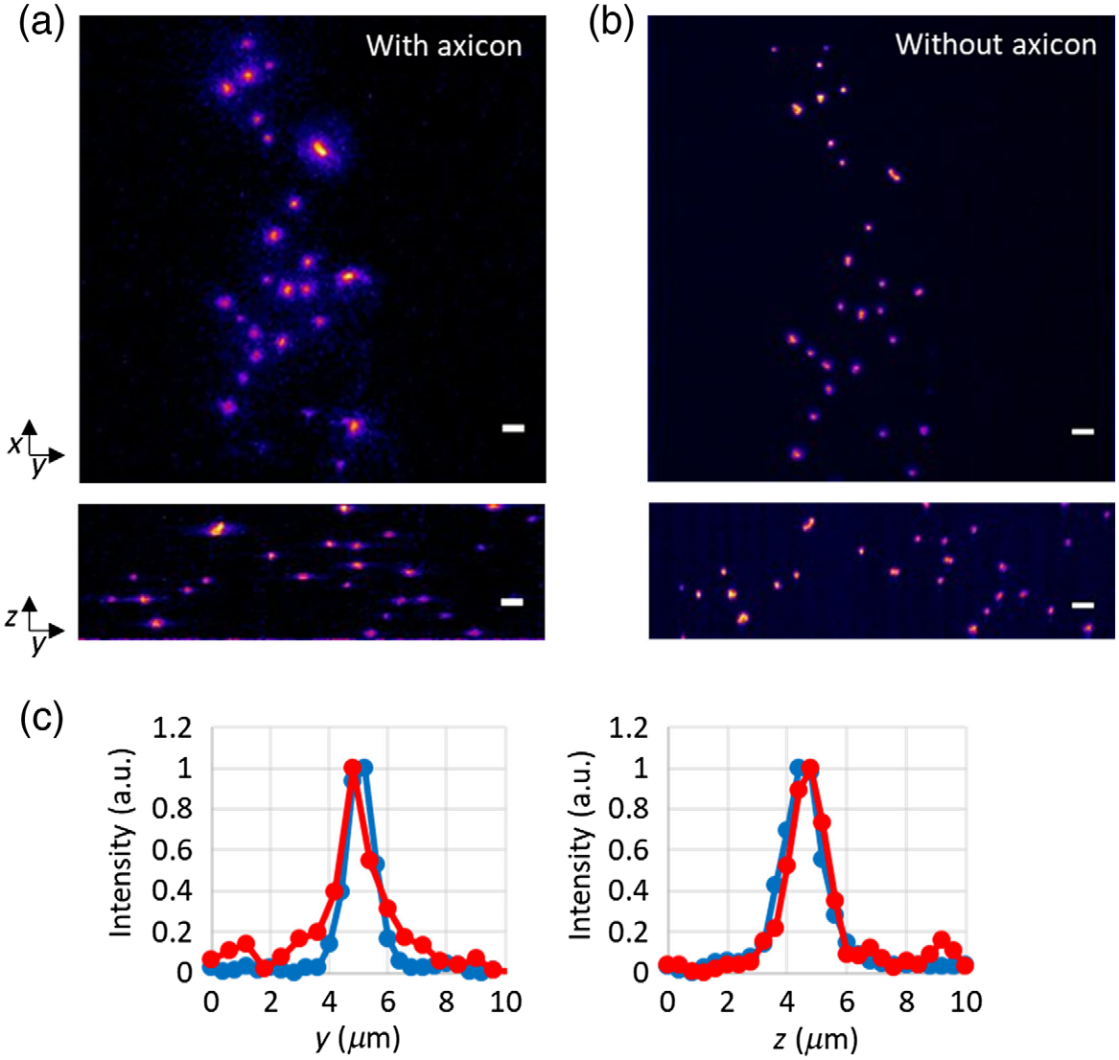
Imaging fluorescent beads by 2p LSFM (a) with and (b) without axicon imaging. (c) Normalized intensity line profile through a fluorescent bead. Red: with axicon imaging. Blue: without axicon imaging. Scale bars: 5  μm.

When taking advantage of LSFM, the axial resolution becomes dominated by the thickness of the light sheet. Although the inclusion of an axicon in the imaging part of 2p LSFM diminishes its axial resolution, the effective PSF still provides excellent optical sectioning capability. We used fluorescent microbeads embedded in agar gel to assess the performance of 2p LSFM with axicon imaging. One microliter of 1-μm fluorescent beads was embedded in 1% low melting point agarose. The mix was spotted on a 5-mm coverslip to create a sample with appropriate volume. Figures 2(a) and 2(b) show the lateral and axial views of the fluorescent beads imaged using our 2p LSFM system with and without axicon imaging. The lateral view was obtained from a maximum intensity projection of a volume image (75 slices, 0.4  μm sections). When using the 2p light sheet without axicon imaging, the scanning of the excitation plane and the detection objective must be synchronized across the volume sample in order to keep each image in focus. However, when using axicon imaging in 2p LSFM, the detection objective is immobile and only scans the excitation plane across the sample. Whole 3D volume images can be obtained. The normalized intensity line profile through one of the beads also demonstrates the isotropic resolution in 2p LSFM with axicon imaging, as shown in Fig. 2(c). We also measured FWHM of five beads and found that lateral and axial FWHM with axicon imaging were almost identical (x, y: 1.12μm and z: 1.34  μm), had uniform cross imaging depth, and were similar to the lateral and axial FWHMs of 2p LSFM without axicon imaging (x, y: 1.03  μm and z: 1.34  μm). We also compared the fluorescence intensity of beads imaged by 2p LSFM with axicon and found that there is an 85% reduction in detected photons when compared to standard imaging. Although images with a low signal-to-noise ratio are difficult to analyze, the emerging computational methods such as deep learning can improve the image quality as well as image resolution.^43^

To examine the capability of 2p LSFM with axicon imaging for fast 3D volumetric imaging, we performed *in vivo* imaging of highly dynamic physiological processes, such as a beating heart and blood flow in zebrafish. For this purpose, we used the Tg(fli1a:EGFP; gata1:DsRed) zebrafish line provided by the Taiwan Zebrafish Core Facility at NHRI and Taiwan Zebrafish Core Facility at Academia Sinica. Embryos were raised at 28.5°C and treated with egg water containing 0.003% 1-phenyl-2-thiourea beginning at 24 h postfertilization. For imaging, embryos were anesthetized with 0.01% Tricaine (Sigma-Aldrich) solution and embedded in 1% low melting point agarose on a 5 mm coverslip. To perform 2p LSFM, we set the laser wavelength to 950 and 1040 nm for excitation of GFP and DsRed, respectively. The laser power measured at the back aperture of the excitation objective was set below 200 mW in order to prevent photodamage in the sample.^44^

**Fig. 3:**
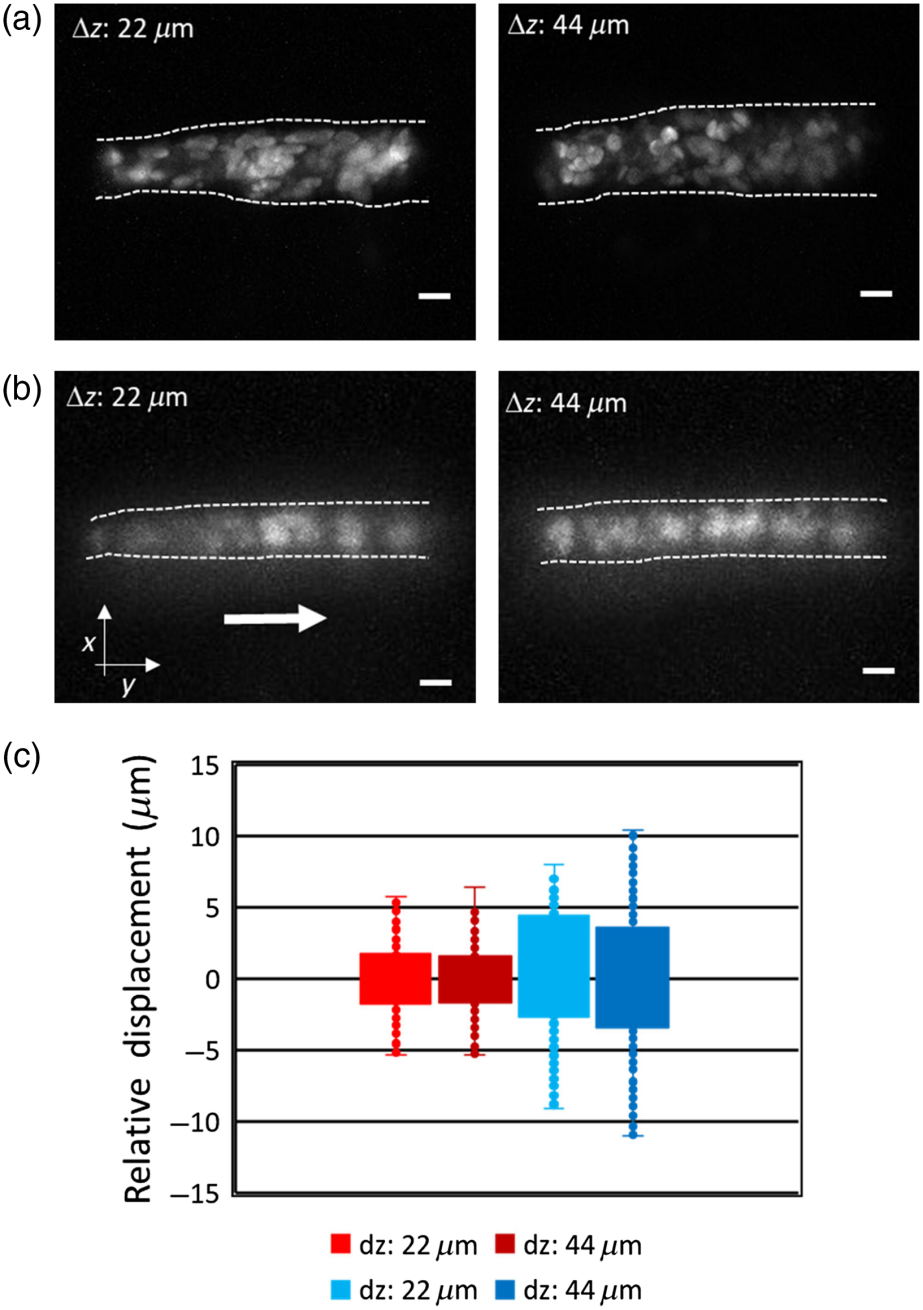
The maximum intensity projection of a volume image (10 slices) of RBCs in Tg(fli1a:EGFP; gata1:DsRed) zebrafish larva over two volume thicknesses using 2p LSFM (a) without and (b) with axicon imaging. (c) Boxplot of RBCs displacement in x axis using 2p LSFM with (red and dark red) and without (blue and dark blue) axicon imaging over 22  μm (red and blue) and 44  μm (dark red and dark blue) volume thickness, respectively. Arrow indicates the blood flow in the y direction. Scale bar: 10  μm.

As shown in Figs. 3(a) and 3(b), we tracked the rapid dynamics of red blood cells (RBCs) in a 3-dpf zebrafish larva using 2p LSFM with and without axicon imaging at a volume acquisition rate of 30 Hz over two volume thicknesses (10 slices), 100  μm×100  μm×22  μm and 44  μm, respectively. With conventional LSFM, the detection objective must be moved in the z direction to achieve 3D imaging, and this movement may severely disrupt the imaging and negatively affect the sample. In Fig. 3(a), the maximum projection images of RBCs using standard 2p LSFM show RBC flow across a vascular cross section with a diameter of 20  μm. In fact, the large diameter of the cross section is due to displacement caused by the movement of the detection objective. As shown in Fig. 3(b), when using 2p LSFM with axicon imaging, the detection objective remains still and the cross-sectional diameter of RBCs flow is only about 10  μm.

Figure 3(c) shows a comparison of the RBC displacement relative to their average position along the x axis between imaging two volume thicknesses using 2p LSFM with and without axicon imaging. We found that the RBC displacement increases as the detection objective scans larger volume thicknesses. These results indicate that the objective scan mode is prone to instability and limits the imaging volume acquisition rate 3D imaging by LSFM. In contrast to the objective scan 3D imaging, 2p LSFM with axicon imaging does not suffer from artifacts induced by objective movement and provides faster 3D imaging capability.

**Fig. 4:**
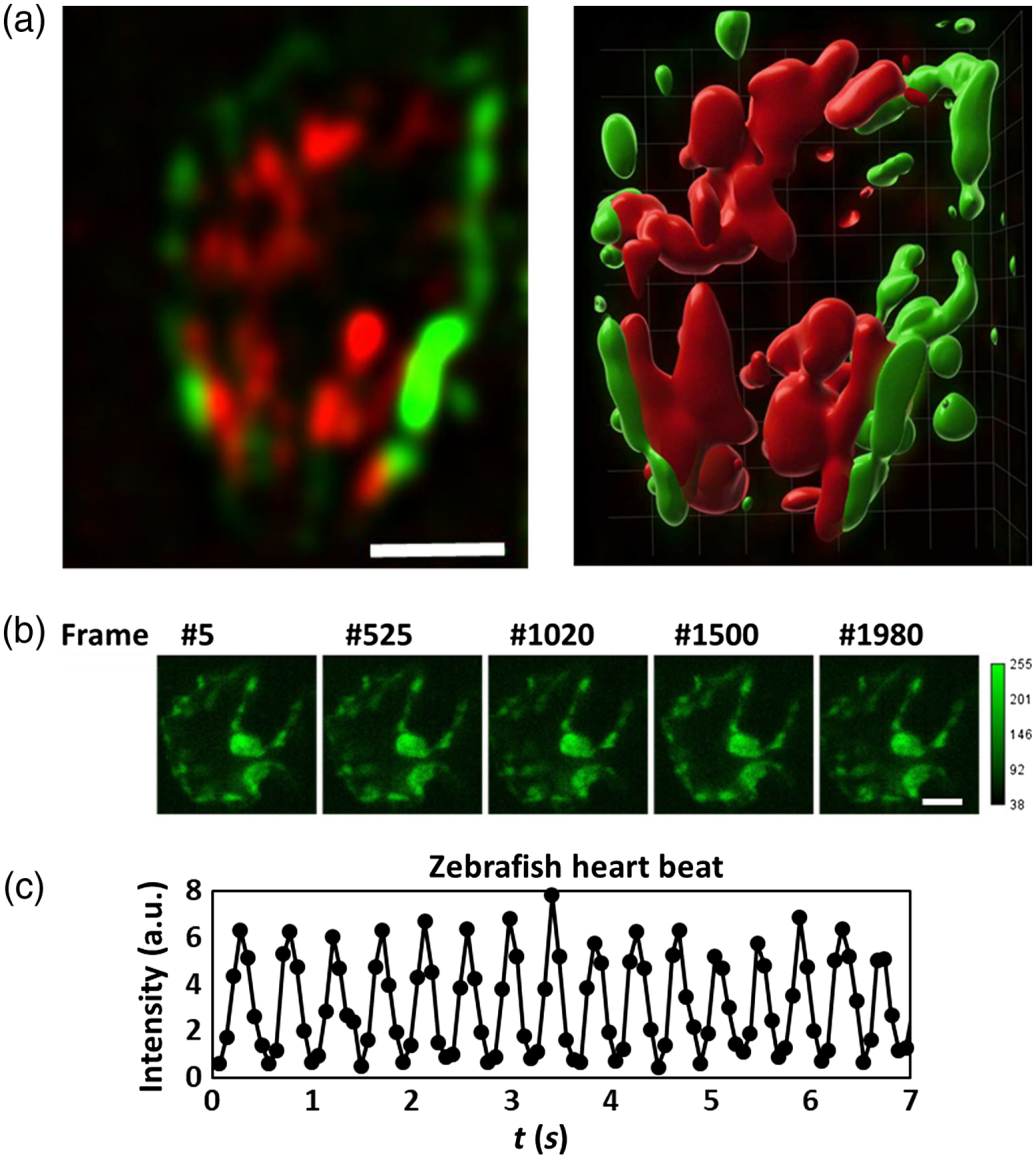
Two-color 2p LSFM with axicon imaging of a zebrafish heart beating. (a) A slice of a volume image and its 3D rendering image (10 slices; 100  μm×100  μm×20  μm) recorded at 10 volumes per second. The transgenic zebrafish Tg(fli1a:EGFP; gata1:DsRed) expressed EGFP in the vascular endothelium and Dsred in RBCs. (b) Representative time-lapse images of a zebrafish heart and (c) zebrafish beating heart dynamics. EGFP fluorescence signal was recorded within an area of the heart as a function of time. The measured heartbeat was 2.2 Hz at 20°C. Scale bar: 30  μm.

Next, to further demonstrate the utility of 2p LSFM with axicon imaging, we recorded heart beating dynamics of embryonic zebrafish that, respectively, expressed EGFP and DsRed in the vascular endothelium and RBCs, as shown in Fig. 4(a). The camera frame rate reached 107 Hz, which allowed us to acquire volumes of 100  μm×100  μm×20  μm at 10 Hz. To evaluate the photodamage effect, we exposed fish embryos to continuous illumination with 200 mW of average power.^44^ No obvious photodamage was detected after long periods of observation by 2p LSFM with axicon imaging, as shown in Fig. 4(b). The heart rate of 2.2 Hz was measured by plotting the fluorescence signals of the GFP-labeled vascular endothelium cells as function of time as shown in Fig. 4(c). This measured heart rate was within the expected range at 20°C.^45^ Moreover, postprocessing synchronization can be used to improve the accuracy of 3D image reconstructions of periodically oscillating objects.^46^ Our system for 2p LSFM with axicon imaging delivered a volume acquisition rate fast enough to capture the dynamic heartbeat of a zebrafish without aliasing.

## 4 Conclusion

In this study, we demonstrated the utility of two-color 2p LSFM with axicon imaging (extended DOF) by capturing rapid processes in a common biological model system, i.e., beating heart and blood cells of larval zebrafish. We also characterized the performance of our system and showed that it can produce a similar image resolution to that of conventional 2p LSFM though there is a reduction in detected photons. Thus the benefits of the light sheet orthogonal configuration can be more fully realized when the axial resolution of axicon imaging is improved. The extended DOF provided by 2p LSFM with axicon imaging is key to achieving fast 3D deep volumetric imaging, as it does not mechanically perturb the sample and still provides isotropic resolution. The volumetric image acquisition rate is therefore only limited by the camera frame rate and photon budgets. The problem of reduced image contrast may be overcome by image postprocessing or selection of a bright fluorescent marker for two-photon excitation. As our results demonstrate, 3D two-photon imaging rates of up to 30 volumes per second are achievable at cellular resolutions suitable for deep tissue imaging. Future applications of this system could involve imaging neuronal activity deep in brain tissue and real-time 3D tracing of neuronal connections.
